# A Machine Learning Classifier for Predicting Stable MCI Patients Using Gene Biomarkers

**DOI:** 10.3390/ijerph19084839

**Published:** 2022-04-15

**Authors:** Run-Hsin Lin, Chia-Chi Wang, Chun-Wei Tung

**Affiliations:** 1 Institute of Biotechnology and Pharmaceutical Research, National Health Research Institutes, Miaoli County 35053, Taiwan; rhlin@nhri.edu.tw; 2 Graduate Institute of Data Science, College of Management, Taipei Medical University, Taipei 10675, Taiwan; 3 Department and Graduate Institute of Veterinary Medicine, School of Veterinary Medicine, National Taiwan University, Taipei 10617, Taiwan; ccwang@ntu.edu.tw

**Keywords:** random forest, gene biomarkers, feature selection, mild cognitive impairment, Alzheimer’s disease

## Abstract

Alzheimer’s disease (AD) is a neurodegenerative disorder with an insidious onset and irreversible condition. Patients with mild cognitive impairment (MCI) are at high risk of converting to AD. Early diagnosis of unstable MCI patients is therefore vital for slowing the progression to AD. However, current diagnostic methods are either highly invasive or expensive, preventing their wide applications. Developing low-invasive and cost-efficient screening methods is desirable as the first-tier approach for identifying unstable MCI patients or excluding stable MCI patients. This study developed feature selection and machine learning algorithms to identify blood-sample gene biomarkers for predicting stable MCI patients. Two datasets obtained from the Alzheimer’s Disease Neuroimaging Initiative (ADNI) database were utilized to conclude 29 genes biomarkers (31 probes) for predicting stable MCI patients. A random forest-based classifier performed well with area under the receiver operating characteristic curve (AUC) values of 0.841 and 0.775 for cross-validation and test datasets, respectively. For patients with a prediction score greater than 0.9, an excellent concordance of 97% was obtained, showing the usefulness of the proposed method for identifying stable MCI patients. In the context of precision medicine, the proposed prediction model is expected to be useful for identifying stable MCI patients and providing medical doctors and patients with new first-tier diagnosis options.

## 1. Introduction

Alzheimer’s disease (AD) is an irreversible neurodegenerative condition characterized by progressive cognitive and memory impairments, which negatively influence an individual’s daily life and the social healthcare system [1]. The total cost of care and treatment related to AD worldwide reaches up to USD 1 trillion per year [2]. As of now, there is no cure for AD, and currently available treatments can only postpone the progression of the disease [3]. Several independent studies indicated that treatment decisions would greatly benefit from early diagnosis, which may delay the progression of AD [2,4,5,6]. In clinical trials, cholinesterase inhibitors (ChEIs) have demonstrated therapeutic benefits for symptoms of cognition, function, and behavior across the disease course [7,8]. These agents are most effective when started early in the disease course and persistently used without treatment gaps.

It was reported that 10.0–15.0% of patients diagnosed with mild cognitive impairment (MCI), an intermediate stage between normal aging (NL) and AD, will develop AD within the first year [9]. After six years of follow-up, approximately 80.0% of MCI patients would convert to AD [1,9]. Therefore, it is of high clinical interest to develop prognostic markers for the early detection of unstable MCI patients [8]. Several early diagnostic methods were developed in recent years with high accuracy. Successful cases include image-based methods of magnetic resonance imaging (MRI) and positron emission tomography (PET), and cerebrospinal fluid (CSF)-based protein biomarkers [10,11,12,13,14,15,16,17,18,19,20,21,22,23,24,25,26,27,28]. However, the image-based methods are expensive, and CSF-based methods require highly invasive lumbar puncture. It is therefore of special interest to develop low-invasive and cost-effective alternatives, such as plasma-based gene biomarkers [29,30,31,32,33]. Owing to the limited number of samples, their generalization ability may require further validation. Furthermore, the accuracy of low-invasive alternatives may not be sufficient for replacing the image- and CSF-based methods. Instead, they can be applied as first-tier screening methods to identify potential unstable MCI patients or exclude stable MCI patients for image- and CSF-based methods. 

This study, therefore, proposed to develop a cost-effective and low-invasive screening method based on plasma gene biomarkers for distinguishing stable and unstable MCI patients. Gene expression and corresponding diagnosis data of study subjects were obtained from the Alzheimer’s Disease Neuroimaging Initiative (ADNI). Machine learning and feature selection algorithms were developed to identify biomarkers for predicting stable MCI patients. The overfitting issues caused by the small number of subjects for biomarkers identification were overcome by incorporating a bigger progression dataset considering any progression from NL and MCI. Based on the two datasets, a common gene set of 31 probes was identified with robust predictive performance. MCI patients with a high stable score can be identified by the developed model with a high accuracy of over 90%. For clinical usage, the developed model offers a new precision diagnosis option with low cost and high efficiency for filtering out MCI patients unlikely to convert to AD. The resource-intensive and invasive diagnostic methods can be reserved for the other patients. It is expected to reduce the overwhelming financial burden of medical care.

## 2. Materials and Methods

### 2.1. Dataset

Gene expression and corresponding diagnosis data of study subjects were obtained from the database of Alzheimer’s Disease Neuroimaging Initiative (ADNI) [4]. The primary goal of ADNI is to support the intervention, prevention, and treatment of AD through the application of new diagnostic methods at the earliest possible stages. Data of serial MRI, PET, biological markers, and clinical and neuropsychological assessment are available at http://adni.loni.usc.edu/ (accessed on 10 June 2020). This study considered only the gene expression data from plasma samples.

Patients with diagnosis data both in the enrollment and second-year follow-up and gene expression data from plasma sampled in the enrollment were first identified. A total of 577 subjects were utilized in this study for biomarker identification and belonged to three groups: NL, MCI, and AD. The numbers of subjects for NL, MCI, and AD are 195, 271, and 112, respectively. Two types of progression models were considered in this study. The first model (named StableMCI model) distinguishes stable MCI patients from unstable MCI patients diagnosed with AD in the second-year follow-up. The second model (named Progression model) considered any progression toward AD, including NL conversion to MCI, NL conversion to AD, and MCI conversion to AD. The development of the StableMCI Model is the primary aim of this study. At the same time, the Progression model was utilized to facilitate the selection of biomarkers for improving the prediction of stable MCI patients. 

Specifically, 358 subjects were identified for developing the Stable MCI model with 46 stable and 312 unstable MCI patients, respectively. There are 577 subjects with 69 unstable and 508 stable patients for developing the Progression model. The gene expression data for each subject was obtained from ADNI and was based on the Affymetrix Human Genome U219 Array with RMA (robust multichip average) preprocessing method [34]. The gene expression data consisting of 49,386 probes was then log2-transformed and z-score normalized to form the feature vector for each subject using the scikit-learn package. Each of the two sets was randomly divided into a training dataset and a test dataset with a ratio of 4:1. The training datasets were utilized for feature selection and model development. Their corresponding test datasets were used for the independent test of the developed models. 

### 2.2. Model Development

Given a large number of features in our dataset, this study applied a rank-based feature selection method to identify the informative features for predicting stable MCI patients. The rank-based method consists of two steps. First, a filter-based method is applied to rank features. The second step applies a wrapper-based method to include top-ranking features until the user-specified convergence condition. Compared to wrapper-based methods, rank-based methods are more computationally efficient and are widely used in previous studies [35,36,37,38,39]. First, chi-square tests were applied to calculate the chi-square value for each probe using the training dataset. Second, the top-n probes were utilized as a feature vector for machine learning, where n ∈ {1, 5, 10, 15, 20, 25, 50, 100, 250, 500, 1000, 49386}. Subsequently, 10-fold cross-validation (10-CV) was conducted to evaluate the area under the receiver operating characteristic curve (AUC) performance for each number of n. Finally, the feature number n giving the highest AUC value was selected as the informative feature for developing a final model. The AUC value ranged from 0 (worse) to 1 (best).

In this study, a random forest algorithm was applied to develop prediction models. The algorithm was developed by Leo Breiman for learning tree ensembles which were used for classification and regression problems [40]. Each decision tree was constructed based on bootstrap samples and randomly selected variables. A majority vote of outputs from all decision trees was applied to make the final prediction. Random forest is shown to be effective and robust for small datasets [41,42,43,44,45] and is considered suitable for this study. The percentage of stable prediction was utilized as the prediction score ranging from 1 (stable) to 0 (unstable). Two parameters of the number of trees and features for constructing a decision tree were set to 100 and log2(total features + 1). The feature selection and random forest algorithms were implemented using the WEKA (Waikato Environment for Knowledge Analysis) package of version 3.8.4. 

## 3. Results and Discussion

### 3.1. Subject Characteristics

The included individuals in this study were identified from ADNI studies. The demographic statistics of the study population are shown in Table 1. A total of 732 subjects (StableMCI: 358, Progression: 577) were included in the present study whose gene expression profiles from blood samples were available for biomarker identification. For StableMCI and Progression datasets, the average chronological ages were 73.5 and 74.4, and the ratios of males were 57.5% and 54.0%, respectively. Both age and gender showed no significant difference in StableMCI and Progression datasets (Table 1).

### 3.2. Identification of Gene Biomarkers 

**Figure 1:**
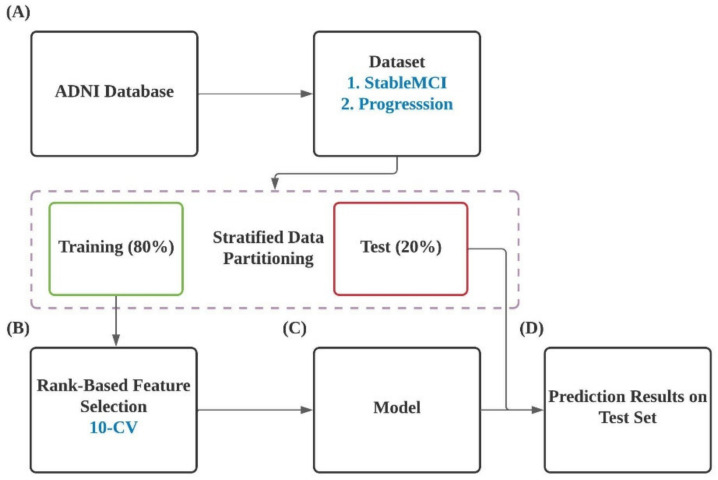
Flow diagram of the present work.

The overall workflow of this study is illustrated in Figure 1. In brief, datasets were extracted from ANDI database and were divided into training and test datasets in a ratio of 4:1 (Figure 1A). A rank-based feature selection algorithm was applied to select informative features with the highest 10-CV performance from 49,386 probes. Prediction models were developed based on the identified informative features and corresponding training dataset. The constructed models were evaluated using the testing dataset.

**Figure 2:**
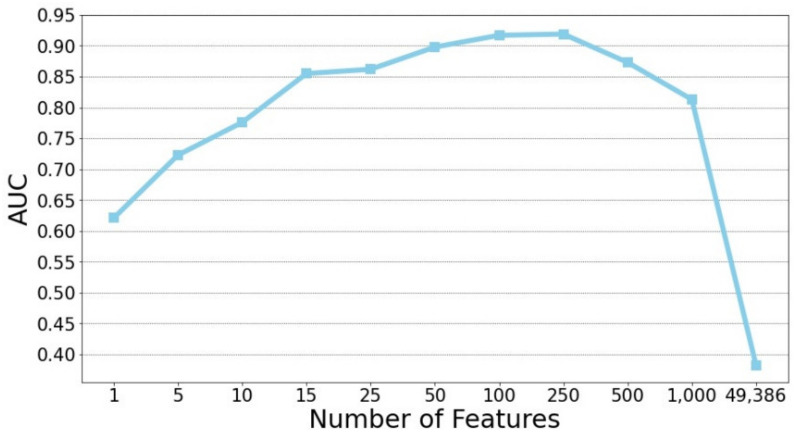
Rank-based feature selection of predictive probes for predicting stable MCI patients.

This study focused on the prediction of stable MCI patients using gene biomarkers. A set of 358 subjects (the StableMCI dataset) were utilized for developing prediction models using the random forest algorithm. First, a chi-square test was applied to rank the probes based on the training dataset. Subsequently, the 10-CV performance of the top n features was evaluated. Since the samples of stable and unstable are unbalanced, the non-parametric AUC measurement was used for evaluating the performance of built models. As shown in Figure 2, the top 250 features gave the highest AUC performance of 0.919. A huge improvement was obtained compared to the AUC value of 0.382 for the classifier using all 49,386 probes. Since the training dataset is small, the feature selection and model development process is likely to be overfitted. Therefore, it is not surprising to see the poor performance of the classifier built on the top 250 features and the whole training dataset with a low test AUC of 0.439 on the test dataset.

While data size is critical for the machine learning task, it is unlikely to obtain a greater number of samples from ADNI in the near future. We therefore proposed to incorporate additional information from the ADNI database. A Progression dataset with 577 subjects was prepared and utilized for feature selection of predictive probes for any progression from NL and MCI to AD. The hypothesis of this study is that true predictive biomarkers for AD should be both useful for predicting stable MCI patients and any progression to AD. The chi-square test was again applied to rank the probes for the Progression dataset, and the AUC performance of the top 250 probes were 0.856 and 0.556 for cross-validation and test, respectively. A total of 31 common probes in the two sets of top 250 probes were then identified. Based on the 31 common probes, reduced cross-validation performance of AUC values of 0.841 and 0.772 was observed for the StableMCI and Progression datasets, respectively. Please note that a few machine learning algorithms, including k-nearest neighbors, naive Bayes, decision tree, and logistic regression, were also evaluated for their prediction performance. However, all of them performed worse than the random forest classifier presented in this study with AUC values less than 0.8. In contrast, the test performance was largely improved, with AUC values of 0.775 and 0.687 for the StableMCI and Progression datasets, respectively. The common probes were considered more robust than the individual probe sets identified from the StableMCI and Progression datasets. The 31 common probes were then utilized to construct the final model for the following analysis.

### 3.3. Independent Test

To evaluate the generalization ability of the StableMCI model built with the 31 common probes, two independent datasets were extracted from ADNI with the same criteria except that the selection of subjects is based on the first-year (StableMCI-1y) and third-year (StableMCI-3y) diagnosis results. To avoid overestimation of prediction performance, subjects included in the StableMCI dataset were excluded from the following analysis. The numbers of unstable and stable subjects are 14 and 130 for StableMCI-1y, and 12 and 53 for StableMCI-3y. Note that the subjects of StableMCI-1y and StableMCI-3y datasets are different from the subjects of the StableMCI dataset. The StableMCI model built with the 31 common probes performed well, with AUC performance of 0.832 and 0.765 for StableMCI-1y and StableMCI-3y, respectively. 

**Figure 3:**
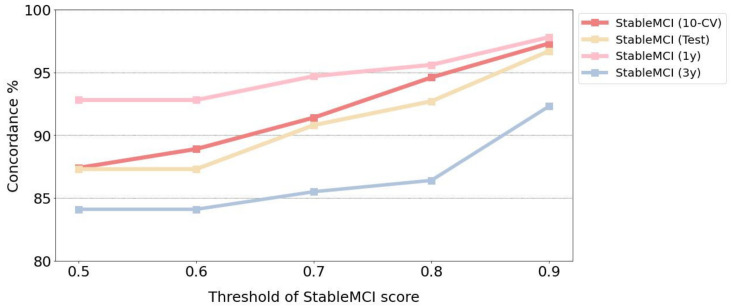
Concordance for various thresholds of the StableMCI score.

The developed machine learning model with good AUC values can be applied to prioritize the MCI patients for progression to AD. To further evaluate the usefulness of the prediction scores made by the developed model, the MCI patients with a StableMCI score greater than thresholds of 0.5, 0.6, 0.7, 0.8, and 0.9 were identified, and the corresponding concordance was calculated. As shown in Figure 3, a higher threshold of the StableMCI score leads to better concordance in all of the datasets of StableMCI, StableMCI-1y, and StableMCI-3y. For the threshold of 0.9, excellent concordance of 97.3% (143/147) and 96.7% (29/30) and coverage of 51.2% and 42.3% were obtained for the 10-CV and test in the StableMCI dataset, respectively. Only four and one patients with a StableMCI score greater than 0.9 were converted to AD. For StableMCI-1y and StableMCI-3y, the same threshold gives excellent concordance of 97.8% (89/91) and 92.3% (48/52), with coverage of 63.2% and 73.2%, respectively.

While good performance was obtained from this study, it is interesting to compare the proposed method with previous studies. We found one study utilizing the same data source and developing a prediction model based on plasma gene biomarkers [29]. In the study, only 66 patients were included in their study. Two partial least square regression models based on 1123 and 225 genes yielded 74% and 77% accuracy using leave-one-out cross-validation. Please note that prediction performance based on only cross-validation without further testing on external datasets could be overestimated. Our model based on only 31 probes is more practical for clinical use and provides better performance than the previous study.

### 3.4. Analysis of the Identified Gene Biomarkers 

To provide a better understanding of the association between the 31 common probes (29 genes) and AD, a literature search and enrichment analysis were conducted. First, the keywords of “Alzheimer’s disease” and “dementia” were utilized along with each gene name to search *PubMed* for identifying published studies with both genes and AD/dementia. As shown in Table 2, 13 and 11 genes were mentioned in the published literature with Alzheimer’s disease and dementia, respectively. Notably, several genes identified in this study are known to be associated with AD. For example, the upregulation of CXCR2 could result in increased production of amyloid-β (Aβ) [46]. CLN3 affects the metabolism of the Aβ protein precursor [47]. Inhibition of CAMKK2 protects hippocampal neurons from the synaptogenic effects of Aβ42 oligomers [48]. The other genes not mentioned in the AD/dementia papers can be further investigated for their roles in AD. 

Some known AD markers, such as TDP43 and tau, were not identified from this study. Our analysis showed both genes of TDP43 and tau are included in the probe list of the studied dataset. However, they were not identified in this study based on the rank-based method. Both algorithmic or biological conditions could be responsible for the exclusion. First, since the dataset is not very big, and a data-driven approach is utilized for the identification of biomarkers, sampling bias could underrate the importance of the two genes. Second, the ranking could be further affected by potential diagnosis errors and experimental variation. Third, the gene expression profiling was done in the enrollment stage, where the gene expression level of the two AD markers may not be affected yet. 

## 4. Conclusions

Early detection of unstable MCI patients is important for better disease management. Current diagnosis methods are, however, highly invasive and expensive. Therefore, this study developed a machine learning model based on gene biomarkers for predicting stable MCI patients. A set of gene probes was first identified from the StableMCI dataset. However, overfitting issues were observed due to the small size of the dataset. An additional Progression dataset was then utilized to derive an additional set of gene probes. A total of 31 common probes were identified with good AUC performance of 0.841 and 0.775 for 10-CV and test, respectively. External tests on unseen subjects of StableMCI-1y and StableMCI-3y datasets achieved good AUC performance of 0.832 and 0.765, respectively. The results showed that the proposed strategy for gene biomarker identification by incorporating the information of any progression to AD is successful.

Among the 29 gene biomarkers (31 probes) identified in this study, 13 and 11 genes were mentioned in the published literature with Alzheimer’s disease and dementia, respectively. Since the expression levels of genes may not be directly related to the inhibitory activity of AD, further experiments are required for studying their roles in AD progression. We also noticed that some known AD markers were not included in the studied dataset. The inclusion of more comprehensive datasets is expected to further improve the study. The investigation of the identified genes without associated papers is also desirable. 

Altogether, the proposed method provides an economic and low-invasive way for the identification of stable MCI patients. While the prediction performance is good, more advanced machine learning algorithms such as XGBoost could be further developed to improve the prediction performance. Based on the proposed method, the identified stable MCI patients will not suffer from invasive or high-cost diagnoses. In addition, medical systems are expected to benefit from the proposed method for resource reallocation, facilitating the precision diagnosis of AD. 

## Figures and Tables

**Table 1:** Characteristics information of datasets for biomarker identification.

	Stablemci Dataset			Progression Dataset		
	Unstable	Stable	*p*-Value	Unstable	Stable	*p*-Value
Number of samples	46	312		69	508	
Age ± years ^1^	74.6 ± 7.5	72.4 ± 8.6	0.52	75.2 ± 7.2	73.6 ± 8.0	0.28
Gender (M/F)	24/22	182/130	0.74	35/34	277/231	0.64

^1^ Continuous variables are presented as mean ± standard deviation, and categorical variables are presented as numbers.

**Table 2:** Co-occurrence analysis of the identified 31 probes (29 genes) and AD/dementia in published literature and corresponding test AUC.

Probes	Gene	Test AUC for Each Probe	Number of Papers Related to AD	Number of Papers Related to Dementia
11750555_a_at	NUP214	0.669	0	0
11715122_at	RAB3D	0.634	0	0
11731423_at	CXCR2	0.625	21	23
11731424_x_at		0.530		
11731425_at		0.502		
11731472_a_at	TMEM70	0.619	1	0
11731473_at		0.611		
11755078_a_at	TADA2B	0.613	0	0
11731379_x_at	MED25	0.599	0	1
11731508_a_at	ZNF649	0.587	0	0
11716944_a_at	YIPF3	0.582	0	0
11731513_at	XPO4	0.581	1	0
11724775_at	FDX1	0.571	0	0
11722278_a_at	SMUG1	0.566	0	0
11731478_x_at	CHMP1B	0.565	1	0
11731477_at		0.473		
11731375_a_at	CAMKK2	0.534	8	7
11731422_s_at	FCGR3A	0.534	0	1
11724369_at	KIAA1644	0.514	0	0
11731479_s_at	TXNDC9	0.513	1	0
11730994_at	S1PR4	0.509	0	0
11722300_a_at	ETS1	0.506	4	4
11731409_at	SLC8A2	0.499	3	2
11723938_s_at	GLOD4	0.487	1	0
11755519_x_at	TGS1	0.446	0	0
11731471_a_at	AKT2	0.444	5	3
11731408_x_at	CLN3	0.434	6	24
11731475_a_at	SFRP4	0.433	0	0
11727610_at	ENSA	0.394	3	1
11731476_x_at	MYL1	0.387	0	1
11731377_s_at	RABL2B || RABL2A	0.376	1	1

## Data Availability

Data used in the preparation of this article were obtained from the Alzheimer’s Disease Neuroimaging Initiative (ADNI) database (https://adni.loni.usc.edu/) (accessed on 10 June 2020).
